# Pristine MXene: In Situ XRD Study of MAX Phase Etching with HCl+LiF Solution

**DOI:** 10.1002/advs.202408448

**Published:** 2024-10-30

**Authors:** Bartosz Gurzęda, Nicolas Boulanger, Andreas Nordenström, Catherine Dejoie, Alexandr V. Talyzin

**Affiliations:** ^1^ Department of Physics Umeå University Umeå S‐90187 Sweden; ^2^ European Synchrotron Radiation Facility (ESRF) ID22 Beamline, 71 Avenue des Martyrs Grenoble 38000 France

**Keywords:** 2D materials, in situ synthesis, MXene, synchrotron radiation, Ti_3_C_2_T_x_, X‐ray diffraction

## Abstract

Many applications are suggested for Ti‐MXene motivating strong interest in studies of Ti_3_C_2_T_x_ synthesis by solution‐based methods. However, so far only ex situ studies of the synthesis are performed, mostly due to the difficulty of handling HF‐based solutions. Here the first time‐resolved in situ synchrotron radiation X‐ray Diffraction study of MXene synthesis performed using a plastic capillary‐size reaction cell directly in HF solution is reported. This study provides the first report on the structure of “pristine MXene” formed by Ti_3_AlC_2_ etching with LiF+HCl. The term “pristine” refers to the MXene structure found directly in HF solution. By comparing the interlayer distances of pristine MXene (≈13.5 Å), solvent‐free Li‐intercalated MXene (≈12.2 Å), and Li‐free MXene (≈10.7 Å), it can be concluded that the width of “slit pores” formed by terminated MX layers during the Al etching does not exceed ≈3 Å. The width of these slit pores is a key factor for HF etching of Al within the interlayers. This space constraint explains the slow kinetics of MXene formation in HF‐based synthesis methods. No intermediate phases are observed, suggesting that the crystalline MXene phase is formed by the simultaneous etching of Al and termination of Ti_3_C_2_ layers.

## Graphical abstract

Time‐resolved in situ synchrotron radiation X‐ray Diffraction study of MAX phase etching by LiF+HCl solution reveals never previously observed structure of “pristine MXene” formed directly in HF solution. In situ monitoring of water washing reveals the transformation of the “pristine” phase into the standard MXene. Extremely narrow slit pores of “pristine MXene” explain the slow kinetics of MXene synthesis in HF‐based synthesis methods.

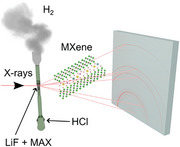

## 1 Introduction

MXenes are materials composed of two‐dimensional (2D) sheets of transition metal carbides.^[^ ^1^ ^]^ These materials attracted significant interest over the past decade^[^ ^2^ ^]^ due to many possible applications, such as energy storage in supercapacitors and batteries,^[^ ^3^, ^4^, ^5^, ^6^ ^]^ electromagnetic shielding, photocatalyst for water splitting,^[^ ^7^ ^]^ removal of pollutants from water,^[^ ^8^ ^]^ optoelectronic devices,^[^ ^9^, ^10^ ^]^ and many others.

MXenes are synthesized by selective etching of the A‐layer (metal atoms) from layered precursors (MAX phases) while retaining the MX layers with a 2D structure. So far, the most studied MXene is Ti_3_C_2_T_X_ produced from Ti_3_AlC_2_ by etching away Al.^[^ ^3^, ^11^ ^]^ The Ti_3_C_2_T_x_ can be delaminated to produce 2D sheets dispersed in suitable solvents, e.g. dimethyl sulfoxide (DMSO).^[^ ^3^, ^12^ ^]^ The 2D Ti_3_C_2_ sheets of Ti‐MXene are terminated by several kinds of functional groups, including, e.g., fluorine, oxygen, and hydroxyls, denoted as “T_x_”. Notably, the properties of MXene and the exact type of 2D flake termination are still somewhat uncertain and depend on synthesis procedures.^[^ ^13^, ^14^, ^15^ ^]^

The earliest method to produce MXene has been to etch aluminum using hydrofluoric acid (HF).^[^ ^11^ ^]^ This method has been later improved by using HF with lithium salts such as LiCl.^[^ ^4^, ^13^ ^]^ The resulting MXene in this method is intercalated by Li, which makes it easier to disperse in polar solvents. A variety of exchangeable cations have been shown to intercalate MXene in salt solutions.^[^ ^3^ ^]^ Significant efforts have been focused on finding MXene synthesis methods to avoid rather toxic and difficult‐to‐handle HF. A variety of etching agents have been proposed over the past decade.^[^ ^14^, ^16^, ^17^ ^]^ For example, one of the most recent and actively studied methods is high‐temperature etching of the MAX phase in molten salts.^[^ ^18^, ^19^ ^]^ However, possibly the most popular in recent years is the synthesis where HF is created “in situ” by using a mixture of LiF and HCl solutions.^[^ ^4^, ^20^, ^21^ ^]^ Notably, the properties of MXene synthesized by the “LiF+HCl” method are significantly different compared to MXene produced only using HF etchant. The presence of exchangeable Li ions intercalated between the MXene layers makes it “clay‐like”^[^ ^4^ ^]^ with swelling properties reminding those of other layered hydrophilic 2D materials, such as graphene oxides.^[^ ^22^, ^23^, ^24^, ^25^ ^]^

Significant interest in optimizing MXene properties for different applications motivates a strong need for studies of the MXene formation mechanism during the process of MAX phase etching. In situ XRD proved to be a powerful method to study MAX phase (Nb_2_GaC) etching in molten salts at elevated temperatures.^[^ ^26^ ^]^ To the best of our knowledge, no structural studies of MXene synthesis have been reported in situ, directly in HF‐based etching solutions. Structural characterization of MXene prepared by HF and LiF+HCl etching methods has been reported so far only for materials studied ex situ after removing the material from the solution and washing it with water. However, both MAX phase and MXene are materials sufficiently well crystalline for recording XRD data directly in solutions.^[^ ^22^, ^23^ ^]^ The main obstacle for in situ experiments seems to be HF which is not only toxic but also dissolves standard glass capillaries typically used to encapsulate liquid samples in XRD experiments.

Here we provide the first time‐resolved in situ study of the synthesis and structure of “pristine MXene”. The term “pristine” refers to the MXene structure found directly in the etching solution, prior to water washing. A special microscopic reaction cell was designed for the experiments, allowing the addition of HCl to the mixture of Ti_3_AlC_2_ and LiF and adapted for using synchrotron radiation XRD to study MXene synthesis directly in the HF solution. The direct transformation of Ti_3_AlC_2_ into never previously reported “pristine MXene” without other intermediate phases was observed at temperatures above ambient. The “pristine MXene” phase is intercalated with HF solution and has a surprisingly narrow interlayer gap width (≈3 Å) sufficient to accommodate only one layer of solution. Therefore, the slow kinetics of MXene formation reported earlier in many studies^[^ ^4^, ^21^ ^]^ is related to the constraints imposed by diffusion of etching agent (HF) and reaction products (AlF_3_ and H_2_) through one‐layer thick “slit pores” formed by Ti_3_C_2_ sheets.

## 2 Results

The absence of in situ data about MXene formation during the etching of the MAX phase is most likely related to technical difficulties associated with handling toxic HF, e.g. because it can dissolve standard glass capillaries used for XRD analysis. Therefore, a special plastic reactor cell was designed for in situ experiments and some modifications of the synthesis procedure were introduced (see experimental section for all the details). Shortly, the time‐resolved data were recorded from ≈1 mm^3^ sample of MAX phase powder pre‐mixed with powder of LiF after adding HCl acid. The reaction of LiF with HCl is expected to produce HF which is then etching Al away from the MAX phase. Analysis of in situ XRD data recorded directly during the etching of the MAX phase demonstrates that “pristine” MXene has the c‐lattice expanded due to swelling in an HF‐based etching solution.

An example of XRD patterns recorded from a sample of mixed powders of MAX phase and LiF before and directly after adding HCl is shown in Figure S1 (Supporting Information). The plastic cell is not entirely amorphous showing some own XRD reflections (Figure S2, Supporting Information). However, no additional reflections due to the cell are found at lower angles where reflections corresponding to the interlayer distance of MXene (001) and precursor MAX phase (002) are expected. A reference XRD pattern recorded from a pure powder sample of the MAX phase is provided in Figure S3 (Supporting Information) showing reflections mostly from (00ℓ) and (h0ℓ) sets.

The pattern was indexed using P6_3_/mmc space group with unit cell parameters a = 3.072(6) Å and c = 18.679(2) Å refined using 22 reflections, and is in good agreement with theoretical data (a = 3.072 Å and c = 18.732 Å).^[^ ^27^ ^]^

**Figure 1:**
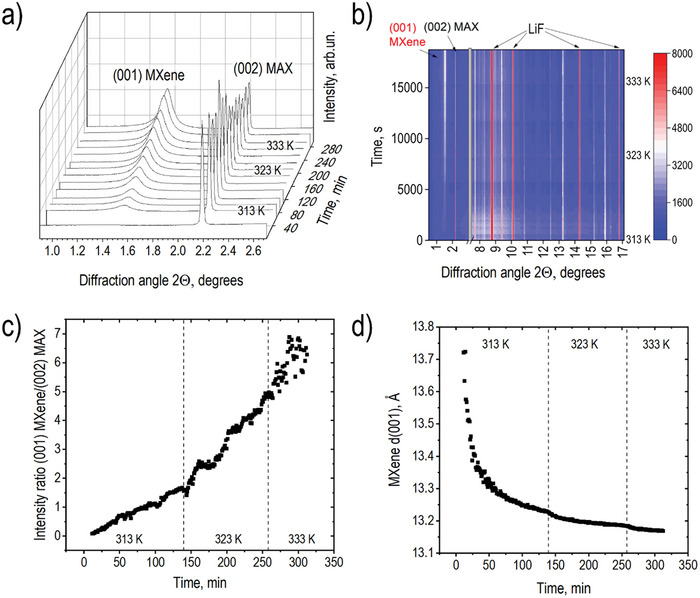
Analysis of XRD data recorded during MXene synthesis at different temperatures. a) Selected XRD patterns recorded in situ from mixed powders of precursors MAX phase and LiF (1:1) after adding HCl (λ = 0.3543 Å). b) “Heat map” showing full range of recorded diffraction angles with break excluding the region of strong reflections from plastic sample holder. c) Integral intensity ratio for (001) MXene/(002) MAX phase reflections as a function of time and temperature. Dashed lines show the moments of temperature change. d) Time dependence of d(001) value for (001) MXene reflection.

The experiment performed with HCl added to MAX + LiF powder mixture at room temperature (≈295 K) showed no MXene formation even after 1.5 h. However, adding HCl to the powder mixture at 313 K resulted in the appearance of the (001) reflection from the “pristine” MXene phase already after ≈15 min of reaction. “Pristine” in this context refers to the MXene structure in a solution‐swollen state prior to water washing. The intensity of this reflection increased over time but rather slowly. Therefore, the temperature of the sample was increased first to 323 K and after 2 h up to 333 K. **Figure** **1**a shows selected XRD patterns in the low angle region showing (001) reflection of MXene increasing in intensity over the time of the experiment while the (002) reflection from precursor weakens. Figure 1b shows a “heat map” for the full recorded diffraction angle range. Analysis of XRD data shows that MXene synthesis is detected solely by the appearance of the (001) and (002) reflections. The rest of the reflections observed at higher angles are due to solid LiF remaining in excess until the end of the experiment and the precursor MAX phase. New reflections, e.g. from the (h0ℓ) set of MXene did not appear indicating an absence of ordering in 2D titanium carbide layers. That is why we index the reflection corresponding to the interlayer distance of MXene as (001), not (002) as in some earlier publications, considering the absence of Ti_3_C_2_ layers ordering. The remaining reflections of the precursor MAX phase showed no shifts during the acid treatment but gradually weakened due to the etching reaction.

Figure 1d shows the evolution of d(001) which appeared first as a broad weak peak around 13.7 Å but decreased to ≈13.2 Å at the end of this experiment. The decrease by 0.5 Å correlating with the smaller broadening of the (001) reflection is likely to be due to a more regular packing of Ti_3_C_2_ layers.

The d(001) corresponds to the average distance between 2D sheets of MXene. The value of interlayer distance found in our in situ experiments is ≈1 Å larger compared to the value typically observed in MXene produced by the same method (LiF+HCl etching) ex situ after water washing (≈12.2 Å) and air drying. However, the d(001) value of ≈13.2 Å observed in our in situ experiments is recorded for MXene in a solution‐immersed state. The liquid phase in our experiments includes HF produced by the reaction of LiF with HCl but also possibly some dissolved LiF and products of the etching reaction (AlF_3_). Therefore, the “pristine” MXene observed in our experiments is found in a state of swelling in this complex solution. The structure of MXene prior to water washing was never previously reported and analyzed. The structure is found to be distinct from the structure of water‐washed MXene as evidenced below.

**Figure 2:**
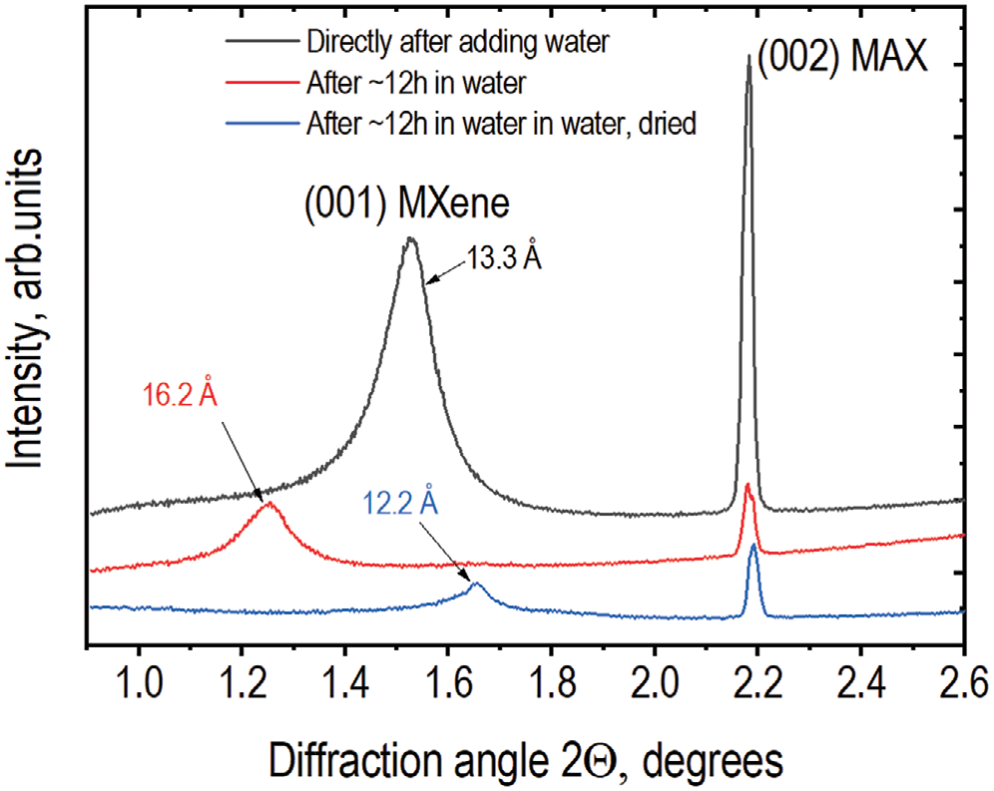
XRD patterns recorded from a sample obtained in the experiment shown in Figure 3 after the replacement of acidic solution with water followed by air drying (λ = 0.3543 Å).

In the next step, the sample was washed with water directly in the plastic capillary. First, water was added to the powder sample using a pipette and the XRD pattern was recorded in water. Next, the sample was left in excess of water for ≈12 h and, after recording a new XRD pattern in the water‐immersed state, it was left to dry on air until all water had evaporated (**Figure** **2**). The final water‐washed dry MXene showed an interlayer distance value of 12.2 Å in good agreement with our own earlier XRD data recorded ex situ^[^ ^22^, ^23^ ^]^ and earlier studies by other groups (12.2–12.4 Å).^[^ ^4^, ^21^, ^24^ ^]^ The XRD pattern recorded from the washed sample and again immersed in water showed d(001) = 16.2 Å corresponding to the water‐swollen state of Li‐intercalated MXene.^[^ ^23^, ^28^ ^]^ Note that only after prolonged water washing the sample was transformed into the standard “clay‐like” Li‐intercalated MXene capable of swelling in water. The LiF reflections were found in this sample even after prolonged water washing since LiF has relatively low solubility in water. Therefore, the swelling of MXene was observed in saturated LiF solution rather than in pure water. The overall decrease in the intensity of the MXene (001) reflection is due to the significantly smaller amount of powder left in the capillary after water washing; most of the material was lost despite the PP mesh being placed around. Note that the position of the (002) reflection of the MAX phase precursor is not affected by washing, drying, and exposure to acidic solution (Figure 2).

**Figure 3:**
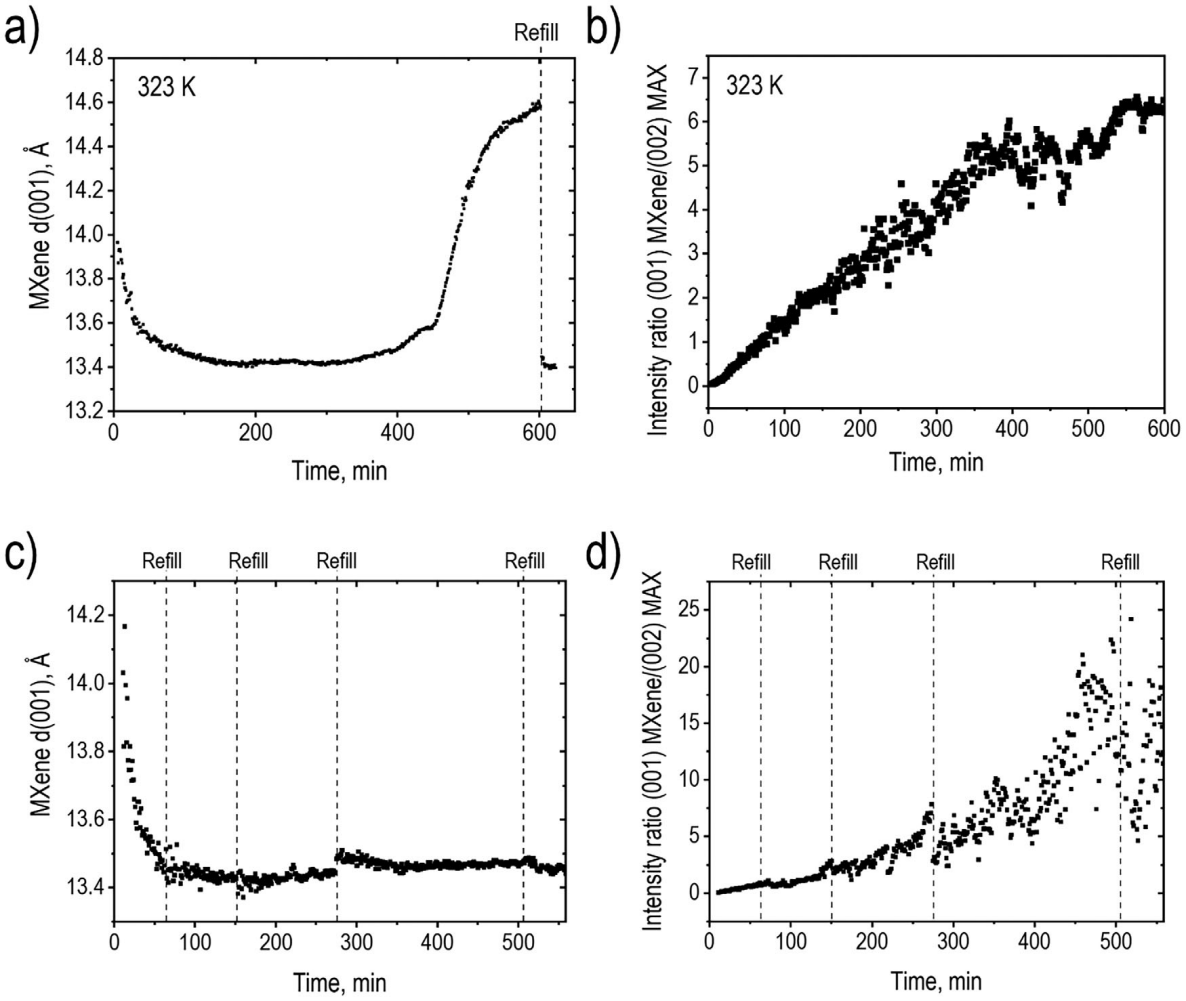
Two experiments with MAX phase etching performed at 323 K. a) MXene d(001) as a function of time after adding HCl. Starting from ≈400 min, effects related to insufficient amount of HF in the solution start to be evident. Refill with HCl was done after 600 min. b) Integral intensity ratio for (001) MXene /(002) MAX reflections versus time for the experiment shown in a). c) MXene d(001) as a function of time‐showing points (dashed lines) when more HCl was added. d) Integral intensity ratio for (001) MXene/(002) MAX reflections versus time for the experiment shown in panel c).

Next experiments were performed with the aim of achieving a complete transformation of the MAX phase into MXene using prolonged etching treatment at 323 K. These experiments were performed using two slightly different procedures. The first experiment was performed using a single addition of HCl to the powder mixture of MAX phase and LiF followed by ≈11 h monitoring of the reaction (**Figure** **3**a,b). However, analysis of the data recorded during the prolonged treatment showed that a single addition of HCl is not sufficient for etching due to the small size of the reaction cell. Similar to the experiment shown in Figure 1, the MXene reflection corresponding to the interlayer distance appeared first ≈13.9 Å. After ≈3 h, this value decreased to ≈13.4 Å and remained almost unchanged during the next 4 h.

However, it started to increase again at the end of the experiment reaching ≈14.5 Å (Figure 3a). The change of d(001) which started after ≈400 min correlates with the slowdown of the increase in MXene peak intensity (Figure 3b). Therefore, we assumed that all HF was consumed after several hours of reaction. This conclusion is evidenced by the d(001) returning back to ≈13.3 Å after refill of the reaction cell with fresh HCl solution (Figure 3a). Complete conversion of all MAX phases into MXene was not achieved in this experiment even after ≈10 h of etching at 323 K, at least partly due to an insufficient amount of HF acid.

Therefore, the second experiment was performed using periodic addition of acid which allowed to achieve a much higher degree of MAX phase conversion to MXene. Note that each refill results in significant turbulence in the powder sample and leads to some change in the absolute intensity of main reflections. Regular refilling with fresh HCl resulted in more stable reaction conditions, as evidenced by d(001) value remaining to be ≈13.5 Å up to the end of the experiment after an initial decrease from 14.2 Å over the first 80 min (Figure 3c). After ≈10 h of etching with 4 HCl refills, an almost complete transformation of MAX phase precursor into MXene was observed with (001) MXene /(002) MAX reflections intensity ratio reaching ≈17 (Figure 3d). Note that large scatter in intensity ratios closer to the end of the experiment is related to spotty diffraction rings and low intensity of (002) MAX phase reflection.

**Figure 4:**
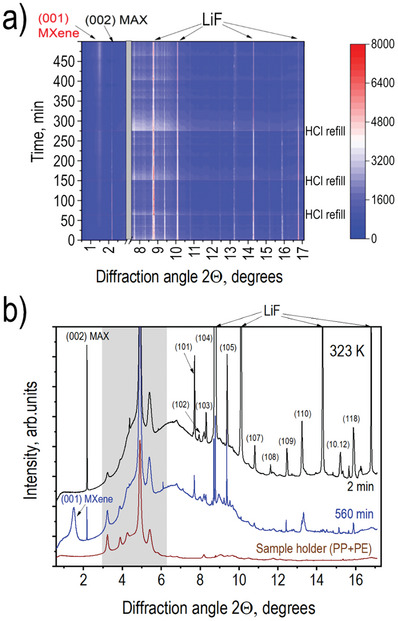
XRD data recorded during the prolonged etching experiment with several refills of reaction cells with fresh HCl. a) Time‐resolved map of XRD patterns recorded during MAX phase etching at 323 K showing time for HCl refills (λ = 0.3543 Å). b) XRD patterns recorded 2 min and 560 min after adding HCl. XRD pattern of material‐free PE capillary with PP mesh is shown as a reference. All reflections in 2 min pattern are indexed according to the composition of the initial powder mixture as LiF (shown by arrows) and Ti_3_AlC_2_. After 560 min the XRD pattern shows (001)‐reflection from MXene. Two very broad background features found in the angle region ≈5–10 degrees are due to the liquid phase.

Similar to the first experiment performed at different temperatures (Figure 1), the reflections corresponding to the ordering of MXene layers did not appear in both experiments performed over a longer etching time at 323 K (see Figures S4 and S5, Supporting Information). Detailed analysis of the XRD pattern recorded at the end of a prolonged experiment with several refills shows a strong decrease in the intensities of LiF reflections, weak reflections from precursor MAX phase, and major (001) reflection from MXene with d‐spacing value of 13.5 Å (**Figure** **4**a,b).

Once again, the XRD patterns do not reveal clearly new MXene reflections other than (001), indicating complete disorder in 2D layers. For example, a new set of reflections from the (h0ℓ) lattice had to appear for MXene due to the increased (compared to precursor MAX phase) c‐unit cell parameter if the terminated Ti_3_C_2_ layers were ordered. We note that several refills resulted in a decrease in the overall intensity of all reflections due to the partial removal of powder material from the reaction cell, making observation of weaker reflections unreliable (also due to the presence of many small features originating from plastic holders). The absence of order is in agreement with earlier studies performed with MXene in the swollen state with the data recorded from samples immersed in polar solvents.^[^ ^22^, ^23^, ^28^, ^29^ ^]^ The XRD patterns of final MXene also do not reveal the formation of the TiC, thus confirming the efficient termination of Ti_3_C_2_ layers by fluorine and oxygen functional groups.

## 3 Discussion

The experiments presented above provide the first insight into the structure of “pristine MXene” formed as a result of Ti_3_AlC_2_ etching with the LiF+HCl method. The name “pristine” in this case reflects the structure of MXene still intercalated with HF solution, not washed with water, and never exposed to air. Our data also provide new insights into the mechanism of MXene formation.

The following simplified reactions are considered for the reaction of Ti_3_AlC_2_ with HF^[^ ^4^ ^]^:

(1) LiF+HCl→HF+LiCl

(2) $$Ti_{3}\mathrm{Al}C_{2}+3\mathrm{HF}\to \mathrm{Al}F_{3}+3/2H_{2}+Ti_{3}C_{2}$$

(3) $$Ti_{3}C_{2}+2\mathrm{HF}\to Ti_{3}C_{2}F_{2}+H_{2}$$

(4) $$Ti_{3}C_{2}+2H_{2}O\to Ti_{3}C_{2}{\left(\mathrm{OH}\right)}_{2}+H_{2}$$

**Figure 5:**
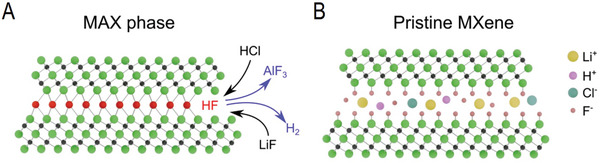
Scheme illustrating the mechanism of MXene formation by reaction of MAX phase with LiF + HCl solution etching away Al layer. a) MAX phase before Al etching by in situ formed HF and b) intercalated Ti_3_C_2_T_x_ MXene.

The addition of HCl to the sample of mixed powders of Ti_3_AlC_2_ and LiF starts the reaction by dissolving LiF and the formation of HF and LiCl (1). The formation of hydrogen bubbles was observed immediately after the addition of HCl, slowing down after a few minutes of the reaction. The reaction obviously starts at the edges of MAX phase flakes and proceeds by removing Al atoms (2) more and more far away from the interlayer entrance points (**Figure** **5**a). The empty space is then immediately filled with the etching solution while the planar surface of Ti_3_C_2_ flakes reacts with both HF (3) and H_2_O (4). The overall formula Ti_3_C_2_T_x_ is used to describe the resulting MXene with T_x_ corresponding to the termination of Ti_3_C_2_ layers by both F and OH groups. The MXene structure is also intercalated by hydrated Li due to exposure to LiCl in solution (Li‐Ti_3_C_2_T_x_).^[^ ^4^ ^]^ According to the results reported in our study, the pristine MXene formed after the removal of Al and termination of 2D layers is still intercalated with HF‐containing solution. The structure of MXene typically reported in the literature is formed after washing of pristine material with water in order to remove HF, while Li is considered to be still intercalated in the “clay‐like” structure which easily swells in water and other polar solvents (Figure 5b).^[^ ^4^, ^22^, ^23^, ^24^ ^]^

Possibly the most important result of our experiment is the observation of a single phase of “pristine MXene” with interlayer distance of 13.9 Å at the initial stages of the reaction. The interlayer distance decreases to ≈13.5 Å and remains the same in prolonged experiments. The reaction steps (2)–(4) were not resolved in our experiments and the crystalline Ti_3_C_2_ phase was not observed. It is likely to be a short‐lived intermediate state that immediately gets converted into fluorine and hydroxyl‐terminated MXene. The reactions (3) and (4) are believed to occur simultaneously leading to the formation of mixed Ti_3_C_2_T_x_ MXene with T_x_ corresponding to all functional groups terminating MXene layers.

It is important to emphasize that the interlayer distance of “pristine MXene” was recorded in our experiments directly in a solution‐immersed state under the condition of swelling. The main parts of this solution are HF and LiCl. The slow reaction with Ti_3_AlC_2_ will also add some AlF_3_ into the liquid phase. The pristine MXene in the solvent‐immersed state shows a complete disorder of 2D layers exhibiting strong (001) reflection with a d‐value corresponding to the distance between Ti_3_C_2_ layers.

Using our data, it is possible to provide an estimate of the size of slit pores (the gap formed between terminated Ti_3_C_2_ layers) that are formed in “pristine MXene” and facilitate further etching. The width of the slit pores is an extremely important parameter of the MXene formation reaction controlling penetration of the etching solution and escape of reaction products (hydrogen and AlF_3_). Considering that the size of MAX phase flakes is at least several µm, the kinetics of MXene formation must be mostly controlled by the diffusion of etching solution from the edges of flakes to the central parts.

The d(001) = 13.5 Å value of interlayer distance was found in this study for pristine MXene immersed in an etching solution. This value needs to be compared to the interlayer distance of solvent‐free MXene. Interlayer distances of mixed Ti_3_C_2_T_x_ fluorine and hydroxyl‐terminated MXene are expected to be equal to ≈10.2–10.7 Å as in experiments with HF etching.^[^ ^11^ ^]^ The experimentally observed value of interlayer distance for the solvent‐free state of Li‐intercalated MXene produced by the “LiF+HCl” method is ≈12.2 Å (Figure 2 and **Table** **1**).

**Table 1:** Values of interlayer distances for standard water‐washed MXene prepared using LiF+HCl method, Li‐free MXene, and pristine MXene (in etching solution). “Δd Pristine” is the difference between inter‐layer distances of Li‐free and Pristine MXene, providing the size of “slit pores”.

	Li‐MXene [dry]	Li‐MXene [in H_2_O]	Li‐free MXene^[^ ^11^ ^]^	Pristine MXene [in HF solution]	Δd Pristine
Inter‐layer distance	≈12.2 Å	≈16.2 Å	≈10.5Å	≈13.5Å	≈3Å

The size of slit pores formed by etching would be surprisingly small if estimated as a difference between the “dry” Li‐intercalated state (≈12.2 Å) and the “pristine” solution‐immersed state (≈13.5 Å). The difference of 1.3 Å is possibly sufficient for the penetration of fluorine anions (≈1.3 Å) but smaller than the size of a water molecule. It would not be sufficient also for the escape of AlF_3_ formed as a product of etching.

Possibly more reasonable is to assume the size of slit pores as the difference between pristine MXene (≈13.5 Å) and Li‐free MXene (≈10.5 Å)^[^ ^11^ ^]^ providing a larger value of ≈3 Å. The width of slit pores in this case is comparable to the size of a water molecule (≈2.5 Å). Nevertheless, even in this estimation, the size of slit pores available for penetration of the etching solution is limited to one monolayer. Therefore, etching is possible only assuming 2D fronts propagating into the depth of interlayers. Simultaneous penetration of the etching solution and removal of AlF_3_ out of interlayers is required for the reaction to proceed.

The size of AlF_3_ molecules is known to be ≈3–5 Å.^[^ ^30^, ^31^ ^]^ Therefore, these molecules must adopt parallel 2D sheet orientation in order to move out of the lattice with 3 Å width “channels”. The size of slit pores is somewhat larger at the initial stages of the etching reaction (≈13.9 Å) indicating that slit pores formed close to the flake edges can be somewhat broader. Note that the (001) reflection of MXene is relatively broad and the ≈13.5 Å value needs to be considered as an average value.

The mechanism of Ti_3_AlC_2_ conversion into MXene was discussed previously in several studies.^[^ ^32^, ^33^ ^]^ However, in the absence of experimental data, much larger interlayer distances (e.g., exceeding the thickness of terminated Ti_3_C_2_ layers) were typically considered in schematic pictures representing the slit pores formed by the removal of Al from the MAX phase and filling these pores by a solution.^[^ ^32^, ^33^ ^]^

The small size of slit pores provides severe limitations to the diffusion of HF inside the interlayers and the diffusion of AlF_3_ out of the MAX phase lattice. Our results indicate that the diffusion in and out of MXene interlayers occurs essentially in a monolayer of an aqueous solution of HF, LiCl, LiF, and AlF_3_.

Hypothetically, the reaction of MXene formation could occur in two steps, first by HF etching of Al and intercalation of Li at the second stage. However, the observation of a single phase of MXene without additional intermediates suggests that a crystalline phase is formed with simultaneous Al etching, termination of Ti_3_C_2_ layers, and Li‐intercalation.

Washing of “pristine” MXene with water should be considered as a separate step in the overall reaction. Removal of HF solution from MXene interlayers by water washing results in a significant expansion of the c‐lattice and an increase of d(001) up to ≈16.2 Å (in a water‐immersed state, before drying) and ≈12.2 Å in a water‐free state. The 4 Å difference between the water‐free and water‐swollen states is larger than the thickness of one water layer (≈2.5 Å). Possibly the value of 4 Å corresponds to random interstratification^[^ ^34^ ^]^ with one and two intercalated water layers. The strong change in properties of MXene suggests that the removal of HF by water washing must be considered as a separate step in the synthesis of “standard” MXene from “pristine” MXene.

It is interesting to note that the ≈13.5 Å interlayer distance seems to be controlled by HF present in the etching solution. A prolonged etching experiment performed without refilling the reaction cell with HCl resulted at some point in an increase of d(001) up to 14.6 Å thus moving toward the value of ≈16.2 Å found in water‐immersed Li‐intercalated MXene after water washing (Figure 2). This effect is likely explained by the relatively small size of the reaction cell and the decrease of HF concentration in the etching solution due to the reaction with the MAX phase. In the absence of HF, more water is inserted around Li‐ions thus explaining the gradual increase of interlayer distance.

Our results demonstrate that the kinetics of the etching reaction is strongly affected by temperature. Formation of MXene was not detected in experiments performed at ambient temperature (295 K) but etching at 323 K over 10 h resulted in a nearly complete transformation of MAX phase precursor into MXene. It should be noted that the increase in temperature results in our experiments also in an increase of HF concentration due to the initial loading of LiF as a solid powder. Solubility of LiF in water increases at higher temperatures thus likely providing higher concentration of HF in the solution.^[^ ^35^ ^]^ Using LiF as a powder in our experiments was motivated by the specifics of the in situ XRD experiments, which require using microscopic amounts of solution (≈1–2 mm^3^). However, we suggest it as a convenient method that could be useful even for bulk ex situ synthesis.

Finally, it is interesting to discuss the implications of the novel results obtained here using in situ XRD for the bulk synthesis of MXene by the LiF+HCl etching method. Experiments presented above demonstrate that the kinetics of MAX phase transformation into MXene can be somewhat improved by an increase in temperature but remains slow even when the reaction is performed at 333 K. Higher temperatures were difficult to test in our experiments due to the use of plastic capillary‐size reaction cells. However, our experiments demonstrate that the slow kinetics of MAX phase etching is likely related mostly to the rather narrow width of the 2D slit pores (≈3 Å) formed by the intercalation of HF into the structure of pristine MXene. Access of HF into MXene interlayers formed in the process of etching and escape of reaction products (AlF_3_ and H_2_) are limited to diffusion in a single layer of HF solution. Therefore, significant improvement in reaction kinetics will be achieved if the width of slit pores formed by MXene interlayers could be increased. This could possibly be achieved in future experiments either by using additional reaction components that would allow expansion of MXene interlayer space directly in the etching solutions or, e.g., by mechanical treatment aimed at delamination of already etched parts of MXene interlayers.

## 4 Conclusion

In situ time‐resolved synchrotron radiation XRD study of Ti_3_AlC_2_ etching with HF produced by the reaction of liquid HCl with LiF powder was performed using plastic capillary‐size cell over prolonged periods of time and at temperatures in the interval 295–333 K. Therefore, we provide the first data about the structure of “pristine MXene” formed directly in process of Al etching reaction and insights into the kinetics of the reaction as a function of temperature.

The results of the experiments can be summarized as follows:

- Experiments performed at temperatures 313 K and higher resulted in the formation of the same “pristine MXene” phase with an interlayer distance of ≈13.5 Å measured directly in the etching solution.
- Prolonged monitoring of the reaction demonstrates a slow increase in the intensity of the (001) MXene reflection and a corresponding decrease in intensity for all MAX phase reflections. The pristine MXene shows complete disorder in the packing of 2D layers.
- A nearly complete transformation of the MAX phase into MXene was observed in an experiment performed at 323 K for ≈10 h with several refills of the reaction cell with fresh HCl. Note that the increase in temperature results in our experiments also in an increase of HF concentration due to temperature‐dependent solubility of LiF.
- The extremely small size of slit pores formed as a result of Al etching (≈3 Å) explains the slow kinetics of MXene formation by HF etching. The size of pores is barely sufficient for the penetration of HF and water into the depths of the interlayers and for the removal of reaction products (H_2_ and AlF_3_).
- Our results suggest that Ti_3_C_2_T_x_ MXene is formed without the formation of intermediate crystalline phases.
- Water washing followed by drying results in the formation of standard MXene with an interlayer distance of ≈12.2 Å. This Li‐intercalated MXene exhibited swelling in water with an increase of d(001) up to 16.2 Å.

Our results indicate that the kinetics of MXene synthesis can possibly be improved in the future if some methods to increase the width of MXene slit pores in the pristine state can be found. The possibility of making in situ analysis of MXene structure in the process of synthesis opens new ways to tune the procedure using experiments with variations of many parameters, like concentrations of solution components, a wider range of temperatures, adding new components into the etching solution to achieve different type of MXene layers termination, etc. Many ideas have been explored so far for modification of MXenes synthesis (see, e.g.,^[^ ^36^, ^37^ ^]^) but the progress in this research can be significantly accelerated by using in situ structural studies.

The microscopic cell designed for our experiments allows us to study the synthesis of many other types of MXenes using a similar type of etching with liquid solutions. Tens of different 2D layered MXenes with a variety of transition metals and thickness of metal carbide layered have been reported by now, but so far synthesis for none of them has been studied in situ.^[^ ^2^ ^]^ The cell also makes it possible to study reactions of MXene synthesis using a variety of other etching agents reported in the literature over the past decade.

## 5 Experimental Section

### Synthesis of Ti_3_C_2_T_x_ MXenes

**Figure 6:**
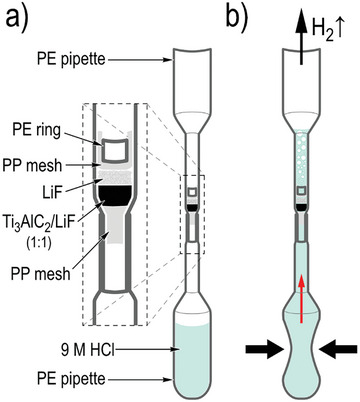
The scheme of the experimental cell is designed for in situ experiments with MXene synthesis.

Lithium fluoride, HCl, MAX phase, and LiCl were purchased from Merck (Germany). A special plastic reaction cell was designed for in situ XRD experiments. Several technical issues make experiments with HF‐solution etching of MAX phases rather challenging, such as the need to use plastic instead of standard glass XRD capillaries (HF dissolves glass), sample movement by gas bubbles formed in the process of reaction, the need for open‐end design to allow gases to escape and small size of samples required for high‐quality data recording and for minimization of HF‐related hazards. After several attempts, we succeeded in designing a microscopic cell that allows safe XRD experiments using synchrotron radiation in transmission geometry. The cell was constructed using two plastic polyethylene (PE) pipettes, one serving as a MAX phase powder holder and the second filled with HCl solution (**Figure** **6**; Figure S6, Supporting Information). Our attempts to directly mix solutions of LiF and HCl over the MAX phase were not successful due to immediate vigorous hydrogen bubbling which forced the sample to move away from the pipette. Therefore, we pre‐mixed the MAX phase with LiF powder (typically in a 1:1 proportion by weight), secured the sample with a polypropylene (PP) mesh, and added HCl using the attached pipette. In this case, it takes some time to dissolve solid LiF before it reacts with HCl thus slowing down the formation of bubbles. Another advantage of using powder LiF is that its reaction with HCl results in the formation of HF only directly around the powder sample, reducing the volume of acid solution to a minimum (≈1–2 mm^3^). The hydrogen bubbles move up, escaping from the opening at the top of the cell while the powder sample is held in place by the PP mesh and PE ring (Figure 6). The XRD images were recorded in transmission geometry using a few second exposures allowing us to monitor the reaction in situ and to record a time‐resolved data set for MXene formation.

The disadvantage of PE pipettes and PP mesh compared to standard glass capillaries is that both are not amorphous materials, exhibiting their own XRD reflections (Figures S1 and S2, Supporting Information). These broad features are especially strong in the angle range of ≈3–6 degrees, but there are also smaller features at higher angles, thus complicating the analysis of full diffraction patterns. A small excess of LiF powder was added on top of the MAX/LiF mixture, to ensure that HF will form in situ during the whole time of the measurement even after additions of fresh HCl.

The powder sample was fixed on both sides with PP mesh to prevent it from moving when the solution was added and H_2_ bubbles started to evolve. The inner diameter of the sample‐holding pipette was 1.8 mm, and the second pipette with a nominal volume of 0.1 mL was attached from the bottom by careful melting of both pipettes using a flame torch and used for adding HCl solution by squeezing it with screws (Figure S6, Supporting Information). The temperature was controlled using nitrogen flow by the Cryojet system. The concentration of HCl used in our experiments was 9 m.

### Characterization

The XRD data were collected at beamline ID22 at the European Synchrotron Radiation Facility using transmission geometry^[^ ^38^ ^]^ using a Perkin Elmer XRD 1611CP3 detector positioned at a distance of 1400 mm from the sample. The X‐ray wavelength was calibrated as 0.354352(4) Å (35 keV) via a NIST standard 640c Si powder. Diffraction patterns were collected during time‐resolved experiments with typical parameters as follows: 10 frames (0.5 s per frame) and 50 s waiting time, thus providing one XRD image in about every minute. Azimuthal integration was carried out using the PyFAI library.^[^ ^39^ ^]^

## Conflict of Interest

The authors declare no conflict of interest.

## Supporting information



Supporting Information

## Data Availability

The data that support the findings of this study are available from the corresponding author upon reasonable request.
