# Supplementary material for: Pristine MXene: In Situ XRD Study of MAX Phase Etching with HCl+LiF Solution
Source: Adv Sci (Weinh). 2024 Oct 30;11(48):2408448. doi: 10.1002/advs.202408448 (PMC11672266; doi:10.1002/advs.202408448)
Supplement: Supplementary file 1 — Supporting Information [file ADVS-11-2408448-s001.docx]

**Supporting information**

Pristine MXene: *In Situ* XRD Study of MAX Phase Etching with HCl+LiF Solution

*Bartosz Gurzęda^1^, Nicolas Boulanger^1^, Andreas Nordenström^1^, Catherine Dejoie^2^, and Alexandr V. Talyzin^1^**

^1^ Department of Physics, Umeå University, Umeå S-90187, Sweden

^2^ European Synchrotron Radiation Facility (ESRF), ID22 Beamline, 71 Avenue des Martyrs, Grenoble 38000, France

**
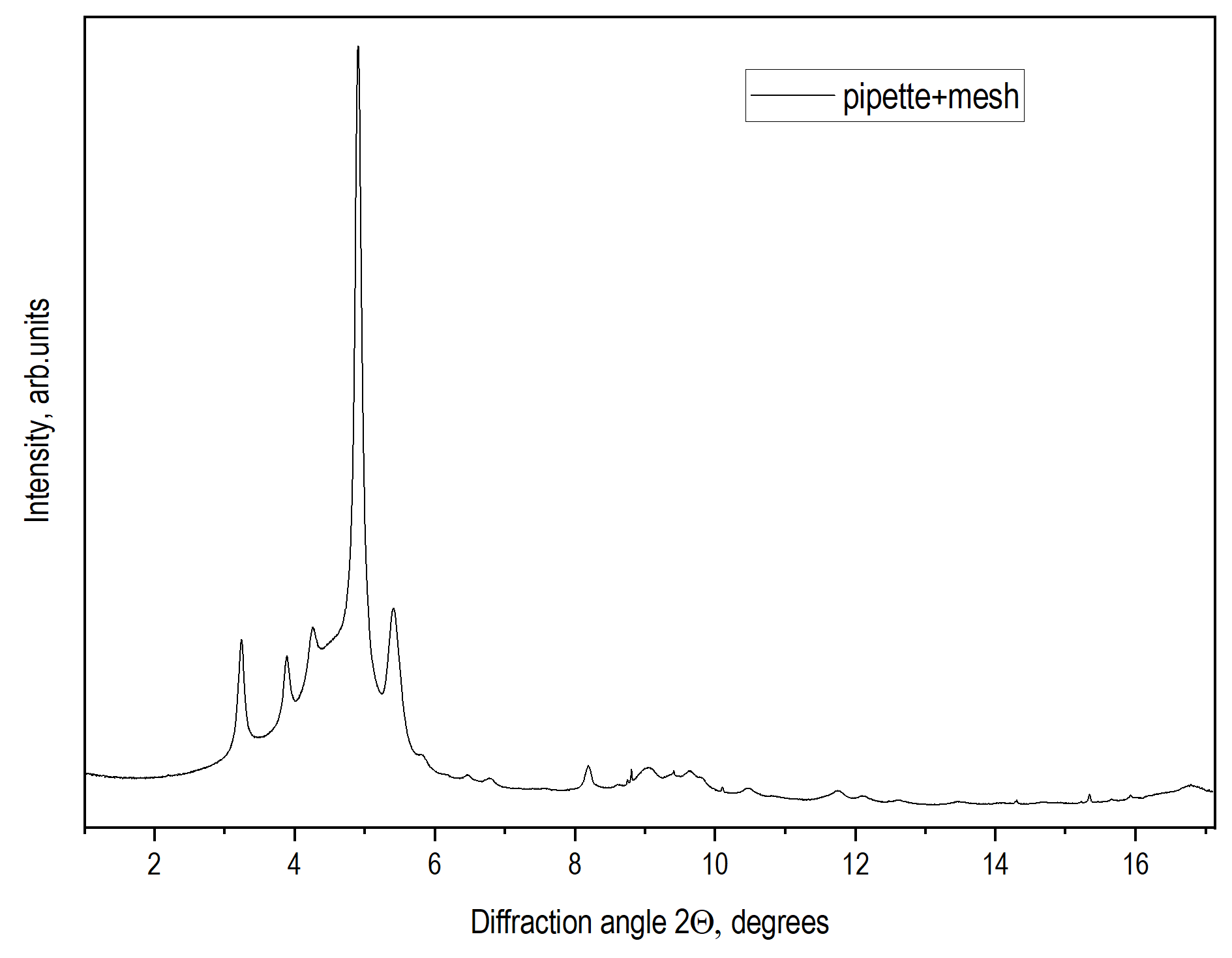
**

**Figure S1.** XRD pattern recorded from powder sample of MAX phase mixed with LiF in 1:1 weight proportion before and after adding HCl solution (λ=0.3543 Å). New broad features appear around 5-10 degrees due to diffraction from liquid phase. The angle region 4-6 degrees has strong reflections from PE pipette and PE mesh. XRD reflections from LiF are shown by arrows. All other sharp reflections are from Ti_3_AlC_2_ precursor. See Figures S2 and S3 for the pattern of material-free PE pipette and PP mesh and indexed XRD pattern of solvent-free Ti_3_AlC_2_ precursor powder.


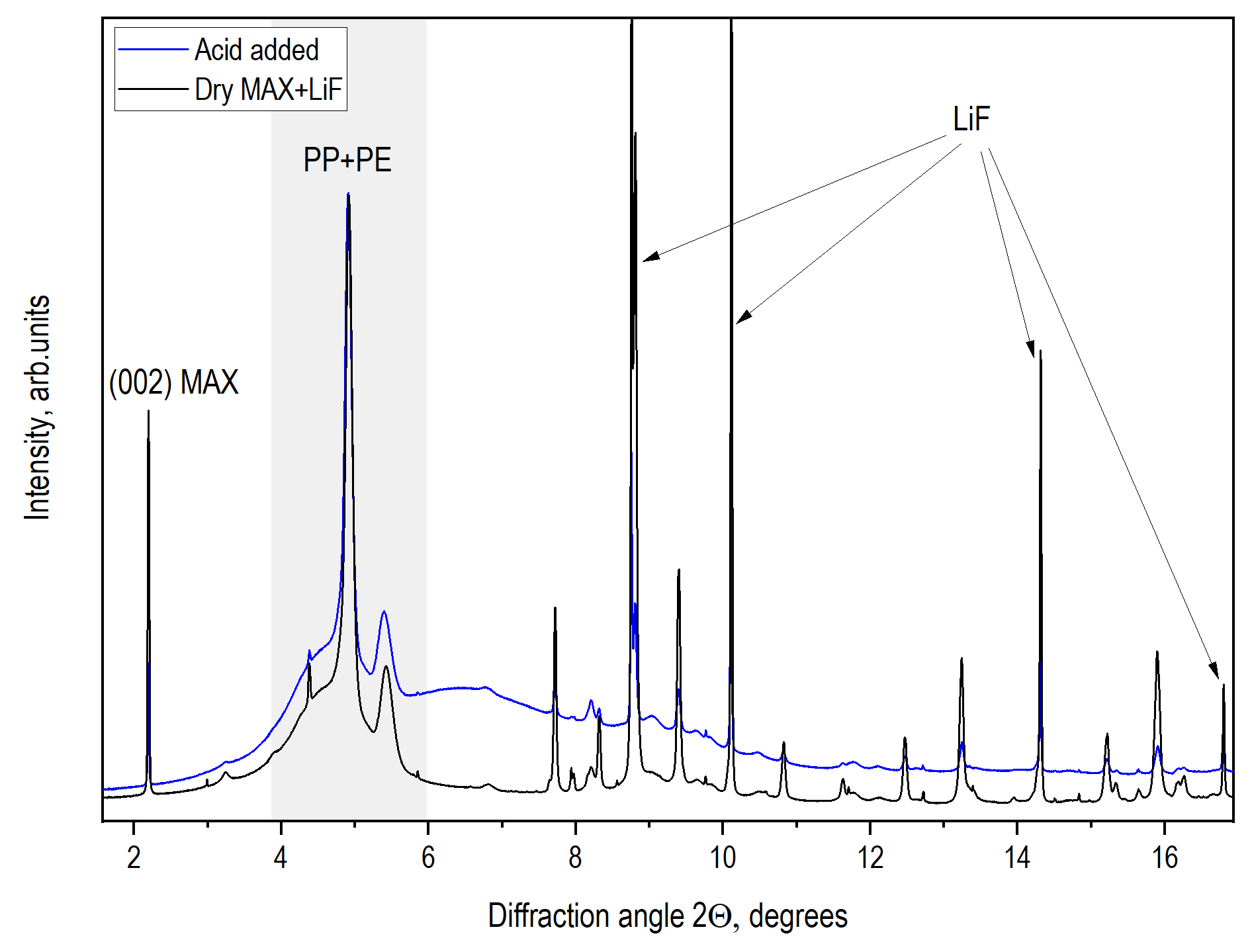


**Figure S2.** XRD pattern recorded form material-free polyethylene pipette filled with polypropylene mesh. It shows rather strong peaks in the angle region from ~3 to ~6 degrees, set of weak features at higher angles, but no peaks at low angle region important for recording typical (002) reflection of MAX phase precursor and (001) reflection of MXene.

**
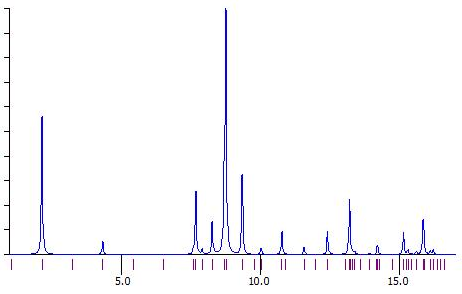
**

**Figure S3.** XRD pattern recorded from precursor MAX phase powder inside of PE pipette. Since MAX phase is well crystalline material, reflections from pipette are relatively weak and not disturbing most of the diffraction angle range. The pattern was indexed using P6_3_/mmc space group with unit cell parameters a=3.072(6) Å and c=18.679(2) Å. Lower panel shows theoretical XRD pattern simulated for Ti_3_AlC_2_ MAX phase using CIF file (mp-3747) thanks to “Material explorer” online database and ref.^1^


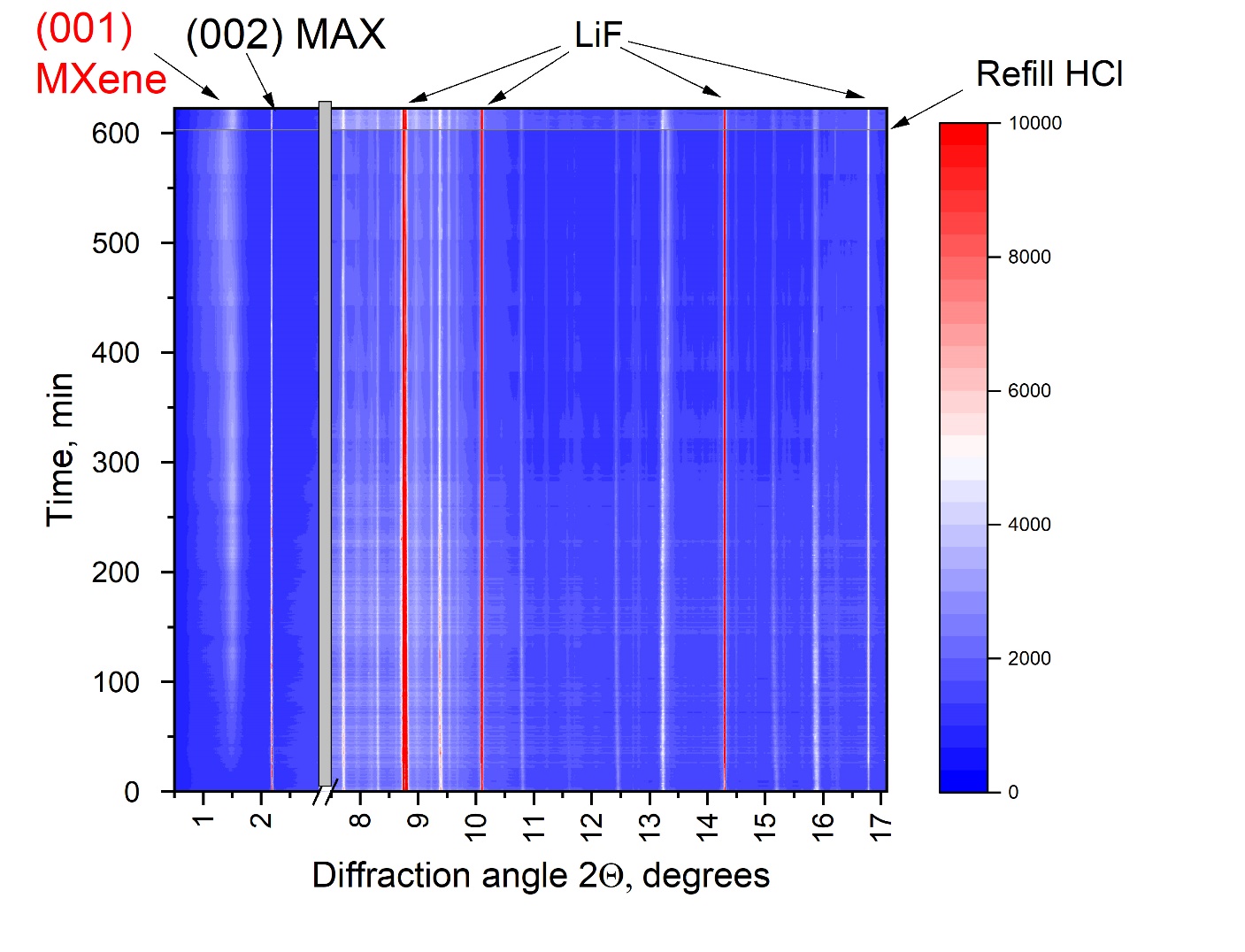


**Figure S4.** Time-resolved map of XRD patterns recoded during MAX phase etching at 323 K performed for ~10 hours without refilling with HCl. Broadening and continuous shift in (002) position to lower angles was observed in this experiment after ~400 min of etching (see Figure 3a, b in the main text). These changes appeared to be related to partial evaporation of solution and depletion from HF due to continuous reaction. Adding fresh HCl restored (002) peak position observed before ~400 min. The angle region 3-7 degrees, most affected by reflections from sample holder (PE pipette+PP mesh), is cut by break. The rest of XRD reflections observed at higher angles are from LiF and MAX phase powders. The figure shows absence of peak shifts for these phases and absence of new reflections corresponding to MAX phase. Complete disorder of MXene layers is common for the material immersed in polar solvents with the structure expanded due to swelling.


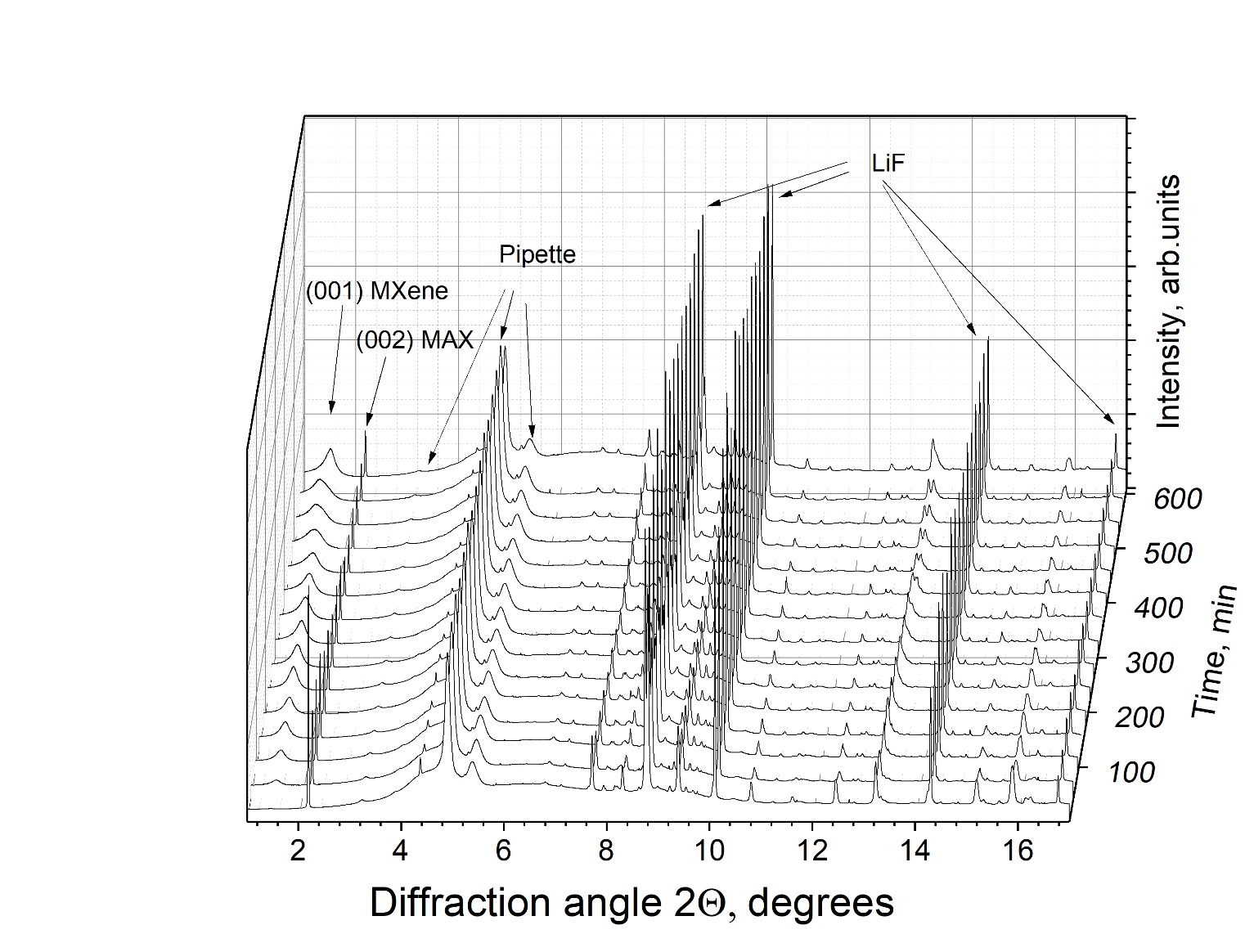


**Figure S5.** Selected XRD patterns recorded during etching MAX phase at 323 K without refills with HCl (same experiment as Figure 3a, b and Figure S4).


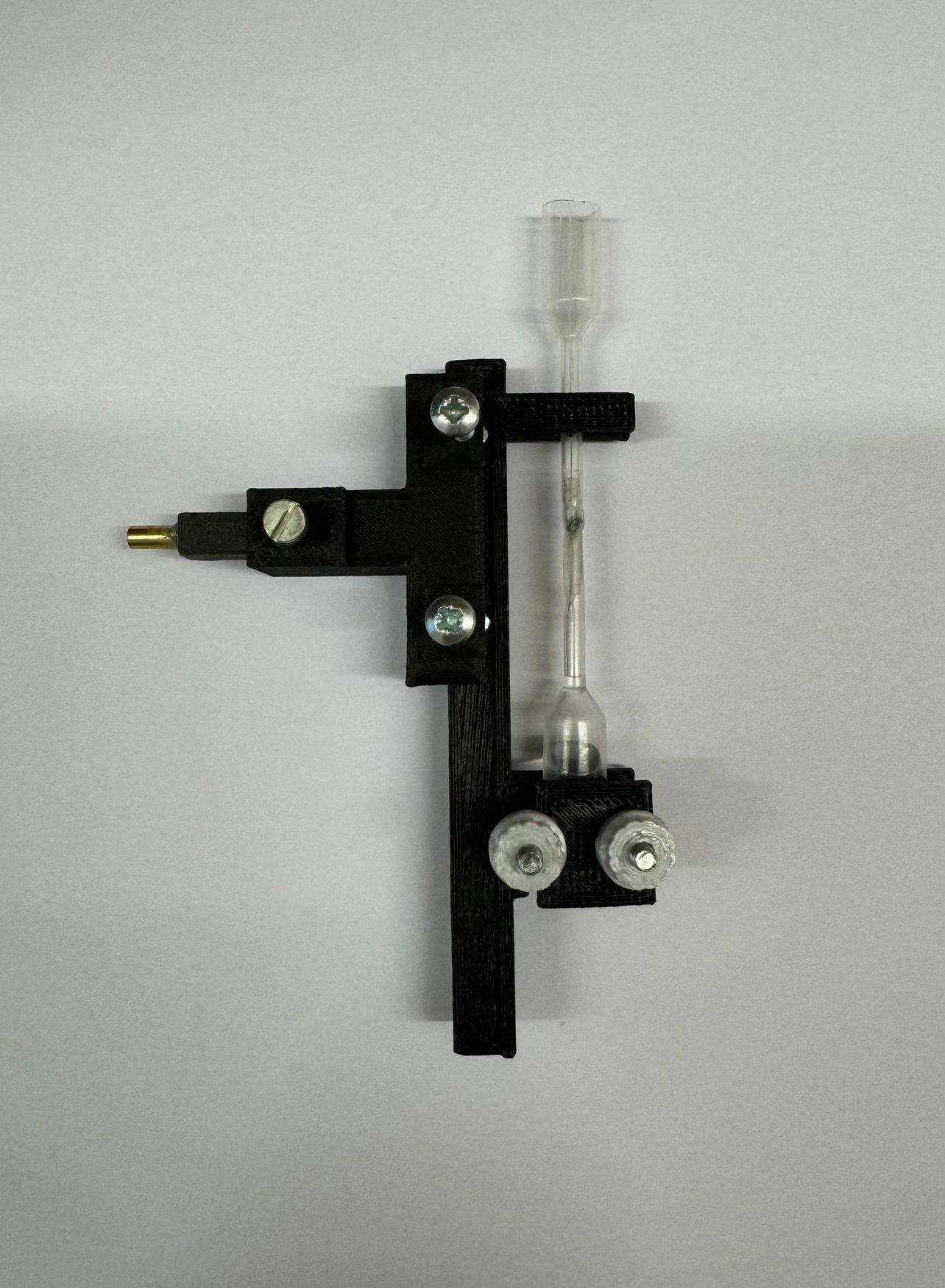


**Figure S6.** Photo of PE experimental cell optimized for in situ XRD studies of MAX phase etching in liquid HF solution.

**References:**

(1) Y. C. Zhou, Y. C.; Wang, X. H.; Sun, Z. M.; Chen S. Q. Electronic and structural properties of the layered ternary carbide Ti3AlC2. *J. Mater. Chem.* **2021**, 11, 2335-2339
